# Marked intestinal trans-differentiation by autoimmune gastritis along with ectopic pancreatic and pulmonary trans-differentiation

**DOI:** 10.1007/s00535-023-02055-x

**Published:** 2023-11-14

**Authors:** Chihiro Takeuchi, Junichi Sato, Nobutake Yamamichi, Natsuko Kageyama-Yahara, Akiko Sasaki, Takemi Akahane, Rika Aoki, Shigemi Nakajima, Masayoshi Ito, Mitsue Yamamichi, Yu-Yu Liu, Nobuyuki Sakuma, Yu Takahashi, Yoshiki Sakaguchi, Yosuke Tsuji, Kouhei Sakurai, Shuta Tomida, Keiko Niimi, Toshikazu Ushijima, Mitsuhiro Fujishiro

**Affiliations:** 1 Department of Gastroenterology, Graduate School of Medicine, The University of Tokyo, Tokyo, 113-8655 Japan; 2 Division of Epigenomics, National Cancer Center Research Institute, Tokyo, Japan; 3 Department of Epigenomics, Institute for Advanced Life Sciences, Hoshi University, Tokyo, Japan; 4 Center for Epidemiology and Preventive Medicine, The University of Tokyo Hospital, Tokyo, Japan; 5 Department of Gastroenterology, Medicine Center, Shonan Kamakura General Hospital, Kanagawa, Japan; 6 Department of Gastroenterology, Nara Medical University, Nara, Japan; 7 Tokushima Health Screening Center, Tokushima, Japan; 8 Department of General Medicine, Japan Community Healthcare Organization Shiga Hospital, Consortium for Community Medicine, Shiga University of Medical Science, Shiga, Japan; 9 Department of Gastroenterology, Yotsuya Medical Cube, Tokyo, Japan; 10 Department of Pathology, Fujita Health University School of Medicine, Aichi, Japan; 11 Center for Comprehensive Genomic Medicine, Okayama University Hospital, Okayama, Japan

**Keywords:** Autoimmune gastritis, Diverse trans-differentiation, Intestinal differentiation, Increased pH, Molecular epidemiology

## Abstract

**Background:**

Autoimmune gastritis (AIG) is a prevalent chronic inflammatory disease with oncogenic potential that causes destruction of parietal cells and severe mucosal atrophy. We aimed to explore the distinctive gene expression profiles, activated signaling pathways, and their underlying mechanisms.

**Methods:**

A comprehensive gene expression analysis was conducted using biopsy specimens from AIG, *Helicobacter pylori*-associated gastritis (HPG), and non-inflammatory normal stomachs. Gastric cancer cell lines were cultured under acidic (pH 6.5) conditions to evaluate changes in gene expression.

**Results:**

Gastric mucosa with AIG had a unique gene expression profile compared with that with HPG and normal mucosa, such as extensively low expression of *ATP4A* and high expression of *GAST* and *PAPPA2*, which are involved in neuroendocrine tumorigenesis. Additionally, the mucosa with AIG and HPG showed the downregulation of stomach-specific genes and upregulation of small intestine-specific genes; however, intestinal trans-differentiation was much more prominent in AIG samples, likely in a CDX-dependent manner. Furthermore, AIG induced ectopic expression of pancreatic digestion-related genes, *PNLIP*, *CEL*, *CTRB1,* and *CTRC*; and a master regulator gene of the lung, *NKX2-1/TTF1* with alveolar fluid secretion-related genes, *SFTPB* and *SFTPC*. Mechanistically, acidic conditions led to the downregulation of master regulator and stemness control genes of small intestine, suggesting that increased environmental pH may cause abnormal intestinal differentiation in the stomach.

**Conclusions:**

AIG induces diverse trans-differentiation in the gastric mucosa, characterized by the transactivation of genes specific to the small intestine, pancreas, and lung. Increased environmental pH owing to AIG may cause abnormal differentiation of the gastric mucosa.

**Supplementary Information:**

The online version contains supplementary material available at 10.1007/s00535-023-02055-x.

## Introduction

Autoimmune gastritis (AIG) is a chronic inflammatory condition of the stomach characterized by an autoimmune response against the parietal cell proton pump H+/K+-adenosine triphosphatase [1–3]. Its prevalence has been reported to be 0.5–19.5% in the general population [3], and as the disease progresses, pernicious anemia due to decreased intrinsic factors and malignant lesions, such as neuroendocrine tumors (NET) and adenocarcinomas, develops [2, 3].

Pathologically, AIG is characterized by the destruction of parietal cells, leading to severe mucosal atrophy in the gastric body and the development of hyperplasia of neuroendocrine cells with the expression of chromogranin and synaptophysin [4, 5]. After long-time chronic inflammation of gastric mucosa, AIG causes intestinal metaplasia similar to *Helicobacter pylori*-associated gastritis (HPG) [2, 3]. However, the molecular mechanisms of chronic inflammation in AIG and HPG are quite different; the former is due to an abnormal autoimmune response in the gastric mucosa, whereas the latter is due to the chronic infection of gram-negative bacteria with virulence factors such as cytotoxin-associated antigen A (Cag A) and vacuolating cytotoxin A (Vac A) [1–3]. AIG-mediated inflammatory cells are known to mostly be autoreactive T cells, whereas those mediated by HPG are predominantly phagocytes, such as macrophages and neutrophils [2, 3].

Despite multiple histological reports on AIG, research on the molecular analysis of AIG is limited [6, 7], and a comprehensive analysis of gene expression using mucosa with AIG is yet to be reported. We previously reported that the gastric mucosa with AIG had a unique DNA methylation profile compared with that of HPG and normal mucosa [6], suggesting the presence of a unique gene expression profile. From AIG-specific gene expression profiles and signaling pathways, we can infer the disease phenotype of AIG, including histological changes, immune responses, and tumorigenesis.

In this study, we aimed to determine whether gastric mucosa with AIG has a specific gene expression profile involved in its histology and chronic inflammation. To address this, a comprehensive analysis of gene expression was performed in gastric mucosa with AIG, that with HPG, and normal mucosa without any inflammation. The potential mechanisms underlying gene expression changes in the gastric mucosa following AIG were also explored.

## Methods

### Study design and tissue sample collection

This was a multicenter study on patients with AIG without *H. pylori* infection, those with HPG, and healthy volunteers who underwent an endoscopic biopsy. The study was approved by the institutional review board of each participating institution and registered at the University Hospital Medical Network (UMIN) on March 1, 2020 (UMIN000039528). All biopsy samples were obtained with written informed consent from all patients.

**Fig. 1:**
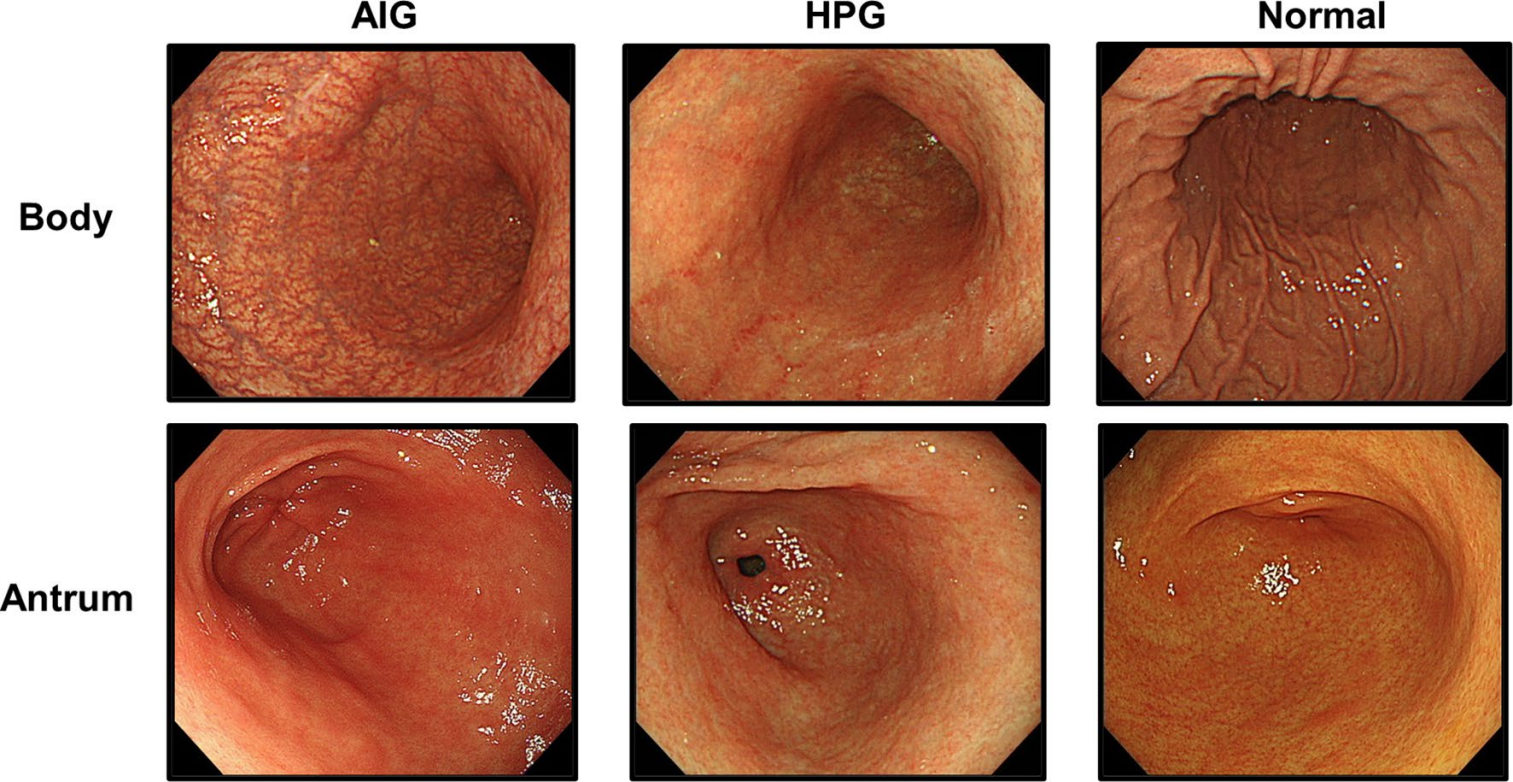
Typical endoscopic views of the gastric body and antrum in cases of autoimmune gastritis (AIG), *H. pylori*-associated gastritis (HPG), and non-inflammatory normal mucosa (normal)

Before enrolment, an endoscopic evaluation was performed. For the AIG and HPG samples, we enrolled only patients with severe open-type atrophy, defined as O-III or O-II in the Kimura–Takemoto classification [8] (Fig. 1). The diagnoses of AIG and HPG were confirmed via blood tests for anti-parietal cell antibody (PCA) and serum anti-*H. pylori* antibody, rapid urease test, or histological *H. pylori* presence, respectively. None of the participants used gastric acid suppressants or had a history of gastrectomy or eradication of *H. pylori*. All biopsy samples were obtained with written informed consent from all patients.

All gastric mucosa samples were endoscopically biopsied from the greater curvature in the middle region of gastric body and were stored in RNAlater (Thermo Fisher Scientific, MA, USA) at – 80 °C.

For a comprehensive analysis of gene expression, 14 AIG samples, 13 HPG samples, and 9 normal samples were used (Table 1). For histological analysis, 10 AIG samples and nine HPG samples were used. For a quantitative real-time RT-PCR, an additional sample set, including 14 AIG samples, 7 HPG samples, and 3 normal samples, was used (Table S1).

**Table 1:** Characteristics of cases used for comprehensive gene expression analyses

Case	Mucosal status	Age	Sex	Anti-HP IgG ( ±)	PG I (ng/ml)	PG II (ng/ml)	PGI/PGII ratio	Anti-PCA titer	Anti-IF antibody	Gastrin (pg/ml)	Vit. B12 (pg/mL)	Ferritin (ng/mL)
JS-A-01	AIG	81	M	−	6.3	11.8	0.5	> 20	+	1800	178	NA
NR-A-01	AIG	70	F	−	3.3	9.3	0.4	> 80	−	6249	273	42
NR-A-02	AIG	58	M	−	3.4	7.6	0.4	> 40	−	2705	64	21
SK-A-01	AIG	59	M	−	NA	NA	NA	> 10	NA	741	140	NA
SK-A-05	AIG	72	M	−	NA	NA	NA	> 10	NA	4085	51	NA
SK-A-06	AIG	87	M	−	NA	NA	NA	> 40	+	3992	NA	NA
TD-A-01	AIG	50	F	−	9.7	6.7	1.4	> 320	NA	2306	475	13
TD-A-02	AIG	78	M	−	7.7	7.5	1.0	> 40	+	3504	538	23
TD-A-04	AIG	73	F	−	14.3	13.5	1.1	> 160	+	12,363	111	19
TD-A-05	AIG	79	F	−	4.3	6.2	0.7	> 20	+	5861	198	66
TD-A-06	AIG	50	F	−	9.7	16.6	0.6	> 160	+	2499	823	14
TD-A-08	AIG	68	M	−	6.6	10.4	0.6	> 40	+	3200	698	18
TD-A-09	AIG	60	M	−	4.3	4.8	0.9	> 20	−	1532	816	94
TS-A-01	AIG	72	M	−	5.1	9.9	0.5	> 80	NA	5946	282	NA
SK-H-02	HPG	61	M	+	NA	NA	NA	NA	NA	NA	NA	NA
SK-H-03	HPG	69	F	+	NA	NA	NA	NA	NA	NA	NA	NA
SK-H-04	HPG	83	F	+	NA	NA	NA	NA	NA	NA	NA	NA
SK-H-05	HPG	76	M	+	NA	NA	NA	NA	NA	351	296	NA
TD-H-01	HPG	70	F	+	NA	NA	NA	NA	NA	876	343	NA
TD-H-02	HPG	69	M	+	NA	NA	NA	NA	NA	NA	NA	NA
TD-H-03	HPG	48	M	+	39.2	11.6	3.4	NA	NA	NA	NA	NA
TD-H-05	HPG	65	M	+	22.5	18.3	1.2	NA	NA	NA	NA	NA
TD-H-07	HPG	86	M	+	NA	NA	NA	NA	NA	NA	NA	NA
TD-H-08	HPG	78	M	+	NA	NA	NA	NA	NA	NA	NA	NA
TD-H-09	HPG	59	M	+	41.1	17	2.4	NA	NA	NA	NA	NA
TD-H-12	HPG	73	M	+	NA	NA	NA	NA	NA	NA	NA	NA
TD-H-14	HPG	56	M	+	55.9	28.3	2.0	NA	NA	NA	NA	NA
JS-N-01	Normal	69	F	−	NA	NA	NA	NA	NA	NA	NA	NA
SK-N-02	Normal	62	M	−	NA	NA	NA	NA	NA	NA	NA	NA
SK-N-03	Normal	76	F	−	NA	NA	NA	NA	NA	NA	NA	NA
TD-N-01	Normal	56	M	−	40.5	7.8	5.2	NA	NA	NA	NA	NA
TD-N-02	Normal	49	F	−	NA	NA	NA	NA	NA	NA	NA	NA
TD-N-03	Normal	74	M	−	NA	NA	NA	NA	NA	NA	NA	NA
TD-N-04	Normal	68	M	−	NA	NA	NA	NA	NA	NA	NA	NA
TD-N-05	Normal	53	M	−	30.1	5.7	5.3	NA	NA	NA	NA	NA
TD-N-06	Normal	40	M	−	49.1	9.6	5.1	NA	NA	NA	NA	NA

*AIG*—autoimmune gastritis, *HPG*—*Helicobacter pylori*-associated gastritis, *PG*—pepsinogen, *F*—female, *M*—male, *PCA*—parietal cell antibody, *IF*—intrinsic factor, *NA*—not available

### Ethics approval

The study using biopsy samples was approved by the Institutional Review Boards of The University of Tokyo (2019173G), Shonan Kamakura General Hospital (1379), Nara Medical University (2554), Tokushima Health Screening Center (713), and Japan Community Healthcare Organization Shiga Hospital (2020–02).

### Comprehensive analysis of gene expression

Total RNA was extracted using an RNeasy Mini Kit (Qiagen, CA, USA). The purity and RNA Integrity Number (RIN) values were assessed using a NanoDrop1000 spectrometer (Thermo Fisher Scientific) and an Agilent 2100 Bioanalyzer (Agilent Technologies, CA, USA). A comprehensive gene expression analysis was conducted using an Agilent SurePrint G3 Human Gene Expression 8 × 60 K v3 Microarray Kit (Agilent Technologies). The obtained data were deposited in the Gene Expression Omnibus (GSE233973) database.

Gene expression data for the tissues were obtained from the Genotype-Tissue Expression Project (GTEx) database (https://www.gtexportal.org/). The mean and standard deviation (SD) of each gene expression level across tissues were calculated, and the top 25 highly expressed genes in a tissue (> mean + 2SD) were identified as tissue-specific genes.

### Gene expression informatics

Quantile normalization was performed using R (version 4.0.5) with the limma package (version 3.46.0) from Bioconductor. Of the 58,201 probes covered by the microarray, 48,858 probes with gene symbols were used for further analysis. The signal intensity values were converted into binary logarithms. Unsupervised hierarchical cluster analysis was performed using R with the Heatplus package (version 2.36.0) from Bioconductor. A volcano plot was constructed using R with the ggplot2 package (version 3.3.6) from CRAN. Gene ontology analysis was conducted using categorical gene set enrichment analysis (GSEA version 4.1.0) (http://software.broadinstitute.org/gsea/) to classify and highlight functionally distinct biological features among the differentially expressed genes; canonical pathway gene sets derived from the KEGG pathway database were used [9]. Tissue enrichment analysis was performed using TissueEnrich (https://tissueenrich.gdcb.iastate.edu/) [10]. The STRING database (https://string-db.org/) [11] was used to map the protein–protein interaction (PPI) network, and the minimum required interaction score was set at high confidence (0.7).

### Quantitative real-time RT-PCR

Reverse transcription was performed using Superscript IV Reverse Transcriptase (Thermo Fisher Scientific). Quantitative RT-PCR was performed with CFX connect Real-Time Detection System (Bio-Rad, CA, USA) using SYBR Green I (Lonza, Basel, Switzerland) and AmpliTaq Gold Polymerase (Thermo Fisher Scientific). A copy number of a gene transcript was obtained by comparison with the amplification of standard DNA samples with known copy numbers and normalized to that of *GAPDH*. The primer sequences for target genes and *GAPDH* are shown in Table S2.

### Histological analysis

Gastric biopsy samples from the middle region of gastric body were obtained, and the differences in the Updated Sydney System (USS) scores for mucosal atrophy and intestinal metaplasia [12] were analyzed between the AIG and HPG samples. USS scores were graded on a scale of 0 to 3 (none, 0; mild, 1; moderate, 2; and severe, 3).

### Immunohistochemistry

Gastric mucosa was freshly obtained and embedded in paraffin following formalin fixation. Sections with a thickness of 2 μm were prepared, and subsequently, deparaffinization, rehydration, and endogenous peroxidase inactivation were performed according to standard procedures. Immunohistochemical staining was performed using antibodies against PNLIP (sc-374612; Santa Cruz Biotechnology, TX, USA), BCL10 (sc-5273; Santa Cruz Biotechnology), NKX2-1/TTF1 (SP141; Roche Diagnostics, Basel, Switzerland), SFTPB (sc-133143; Santa Cruz Biotechnology), and SFTPC (ab90716; Abcam, Cambridge, UK). Stained sections were independently evaluated by two pathologists.

### Cell culture

The AGS cell line was purchased from the American Type Culture Collection (ATCC, Manassas, VA, USA). MKN74, MKN1, and GCIY cell lines were purchased from RIKEN BioResource Center (Ibaraki, Japan). To prepare the acidic culture medium (pH 6.5), Dulbecco’s modified Eagle’s medium (DMEM) with high glucose (Sigma-Aldrich, MO, USA) was supplemented with 10 mM PIPES. For the preparation of control culture medium (pH 8.0), DMEM with high glucose and NaHCO_3_ (FUJIFILM Wako, Osaka, Japan) was supplemented with 10 mM HEPES. These media were supplemented with 10% fetal bovine serum (FBS) and 1% penicillin/streptomycin (Thermo Fisher Scientific) and were adjusted to their respective pH at 37 °C. The pH of the medium was monitored before and after incubation to confirm that it remained constant throughout the experiment.

## Results

The clinical characteristics are shown in Table 1 and S1. All AIG cases showed severe mucosal atrophy predominantly from the gastric body to the fundus and showed high anti-PCA titer (> 10 RU/mL) [13]. No significant differences were noted in age and sex between the AIG, HPG, and normal cases analyzed for comprehensive gene expression analysis (Table S3).

### AIG displayed a unique gene expression profile from HPG in gastric mucosa

**Fig. 2:**
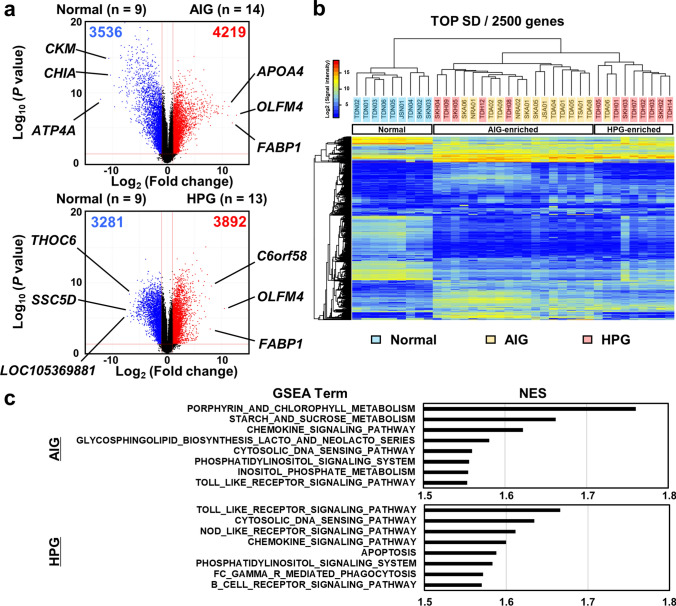
Results of the comprehensive analysis of gene expression among the AIG (*n* = 14), HPG (*n* = 13), and normal samples (*n* = 9). **a** Volcano plot analysis using the fold changes of gene expression levels between the normal and AIG samples, and the normal and HPG samples. The number of gene transcripts with twofold change and a small *P-*value (-log_10_ (*P*-values) > 1.301) was higher in the AIG samples than in the HPG samples. **b** Unsupervised hierarchical cluster analysis using gene expression levels of the total 36 samples. Using the 2500 gene transcripts with the highest standard deviation (TOP SD), the AIG and HPG samples were clearly separated from the normal samples and were further separated into the AIG-enriched cluster and the HPG-enriched cluster. **c** Pathway enrichment analyses conducted via GSEA using the upregulated genes in the AIG and HPG samples. The top eight activated gene sets in the AIG and HPG samples are shown. *NES*—normalized enrichment score

In comprehensive gene expression analyses, several gene transcripts were significantly (*P* < 0.05) upregulated (more than twofold change: 4219 of 48,858) and downregulated (3,536) in AIG samples, whereas 3,892 gene transcripts were upregulated and 3281 were downregulated in HPG samples (Fig. 2a). Unsupervised cluster analysis using 2500 genes with the greatest SD showed that the AIG and HPG samples were clearly separated from the normal mucosa samples and were further separated into the AIG-enriched cluster and the HPG-enriched cluster (Fig. 2b), indicating a unique expression profile of AIG. In addition, *ATP4A* was markedly downregulated (Fig. 2a), indicative of parietal cell damage. *GAST* (gastrin) and *PAPPA2* (pappalysin 2), which have been reported to contribute to the development of NET [14], were highly expressed in the AIG samples (Fig. S1). The top 50 differentially expressed gene transcripts in AIG and HPG samples are listed in Tables S4, S5, S6, and S7.

To explore the pathways upregulated in the gastric mucosa with AIG and HPG, pathway enrichment analyses were conducted using the upregulated genes. With a cutoff *P*-value of 0.01, GSEA identified 14 terms in both AIG and HPG samples (Fig. 2c). The AIG samples were enriched with gene sets associated with small intestinal absorption, such as “porphyrin and chlorophyll metabolism” and “starch and sucrose metabolism,” indicating abnormal intestinal differentiation in gastric mucosa. In contrast, the HPG samples were enriched with many gene sets associated with inflammatory response, such as “Fc-gamma receptor-mediated phagocytosis,” indicating aggressive macrophage- and neutrophil-related inflammation in gastric mucosa with HPG.

### Upregulation of small intestine-specific genes and downregulation of stomach-specific genes induced in gastric mucosa with AIG

**Fig. 3:**
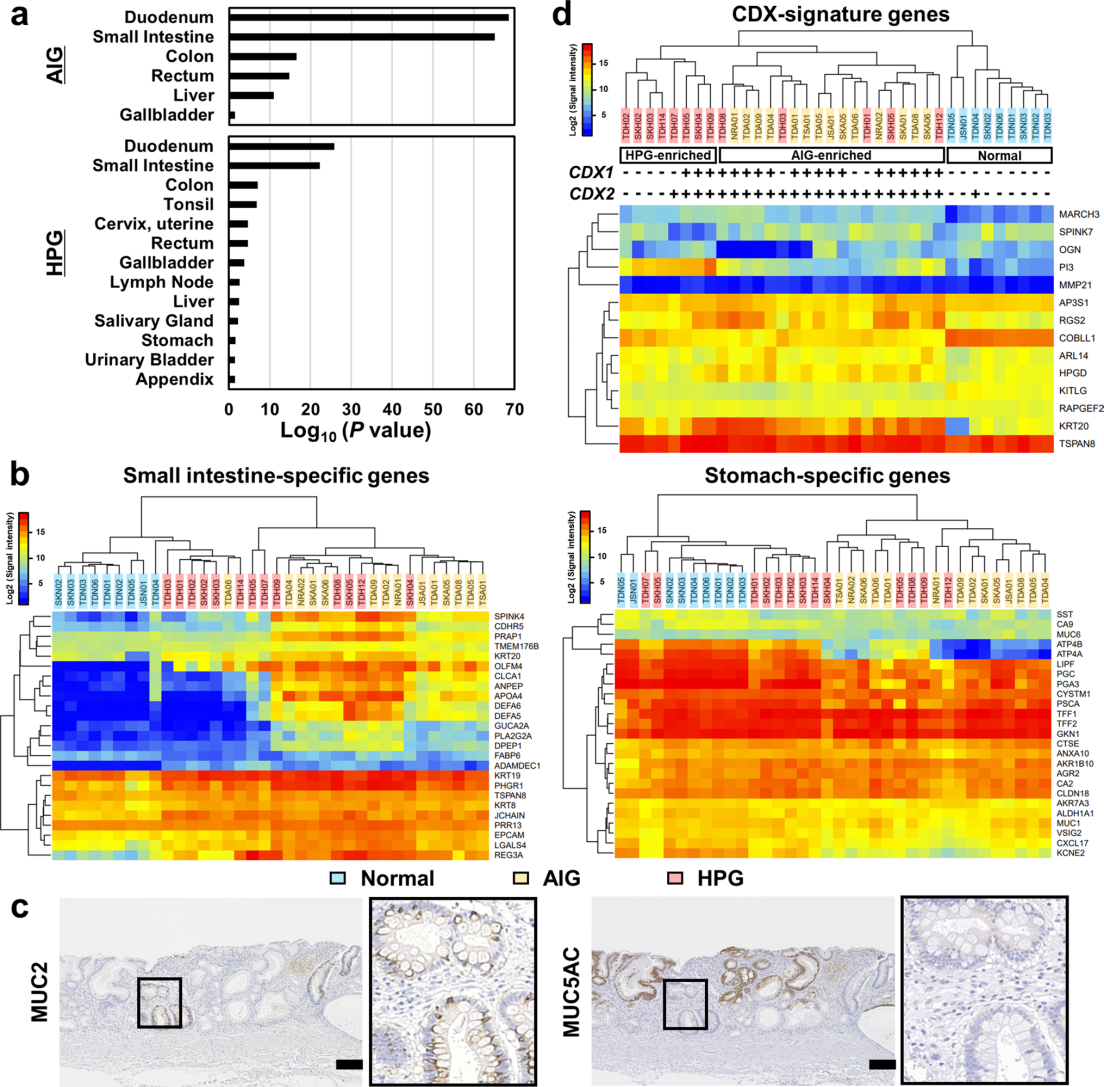
Marked intestinal differentiation in gastric mucosa with AIG. **a** Tissue enrichment analysis using top 50 upregulated genes in gastric mucosa with AIG and HPG. AIG showed higher enrichment of genes specific to the duodenum and the small intestine compared with HPG. **b** Unsupervised hierarchical cluster analysis using gene expression levels of small intestine-specific and stomach-specific genes among the AIG (*n* = 14), HPG (*n* = 13), and normal samples (*n* = 9). The AIG samples were clearly separated from the normal samples using small intestine and stomach-specific genes, while a fraction of the HPG samples were grouped with the normal samples. **c** Immunostaining of MUC2 and MUC5AC using gastric mucosa with AIG. Scale bar: 100 μm. **d** Unsupervised hierarchical cluster analysis using gene expression levels of CDX signature genes, along with *CDX2/1* expression (cutoff signal intensity = 25). The AIG and HPG samples were clearly separated from the normal samples and were further separated into the AIG-enriched cluster and the HPG-enriched cluster. Moreover, all the AIG samples expressed *CDX2*/*1*, while the *CDX*-negative HPG samples showed a unique cluster

Abnormal intestinal differentiation is known to occur in gastric mucosa with HPG [15]. To investigate whether abnormal intestinal differentiation is induced in gastric mucosa with AIG as prominently as in that with HPG, tissue enrichment analysis (TissueEnrich) was performed using the top 50 upregulated genes in gastric mucosa with AIG and HPG. AIG showed higher enrichment of genes specific to the duodenum and small intestine (− log_10_ (*P* value) = 68.5 and 65.2, respectively) than HPG (25.8 and 22.2, respectively) (Fig. 3a). Additionally, mucosal samples were compared by analyzing the expression levels of tissue-specific genes, which were identified as the top 25 highly expressed genes in a tissue with an SD greater than 2SD among all tissue types using the GTEx database. Notably, almost all the AIG samples were clearly separated from the normal samples, in case of small intestine-specific and stomach-specific genes (Tables S8 and S9), whereas a few HPG samples grouped with the normal samples (Fig. 3b), indicating frequent intestinal differentiation in the gastric mucosa with AIG. In the histological analysis using the USS scores, the AIG samples demonstrated a high incidence of intestinal metaplasia (10/11, 90.9%), whereas the HPG samples had a lower incidence (3/7, 42.9%) (Fig. S2a). Moreover, similar to HPG-associated intestinal metaplasia [16], AIG-associated intestinal metaplasia exhibited MUC2 expression but lacked MUC5AC expression (Figs. 3c and S2b).

Caudal-type homeobox (*CDX*) 2 and *CDX1* are master regulator genes for intestinal differentiation [17, 18] and are thought to complement each other [19]. All AIG samples and a half of HPG samples showed high expression levels of *CDX2/1* (Fig. 3d). To examine whether CDX2/1 plays a potential role in AIG-induced transcriptional changes, the gene expression data of AIG, HPG, and normal mucosa were evaluated according to the expression levels of CDX signature genes, which were characterized as the genes affected in gastric cancer cells stably transfected with CDX [20]. The AIG and HPG samples were clearly separated from the normal samples and were further separated into the AIG-enriched cluster and the HPG-enriched cluster (Fig. 3d). Moreover, all AIG samples expressed *CDX2*/*1*, whereas the *CDX*-negative (signal intensity < 25) HPG samples showed a unique cluster. These data indicate that intestinal trans-differentiation may occur in a CDX-dependent manner.

### Gastric mucosa with AIG showed trans-differentiation into pancreas and lung

**Fig. 4:**
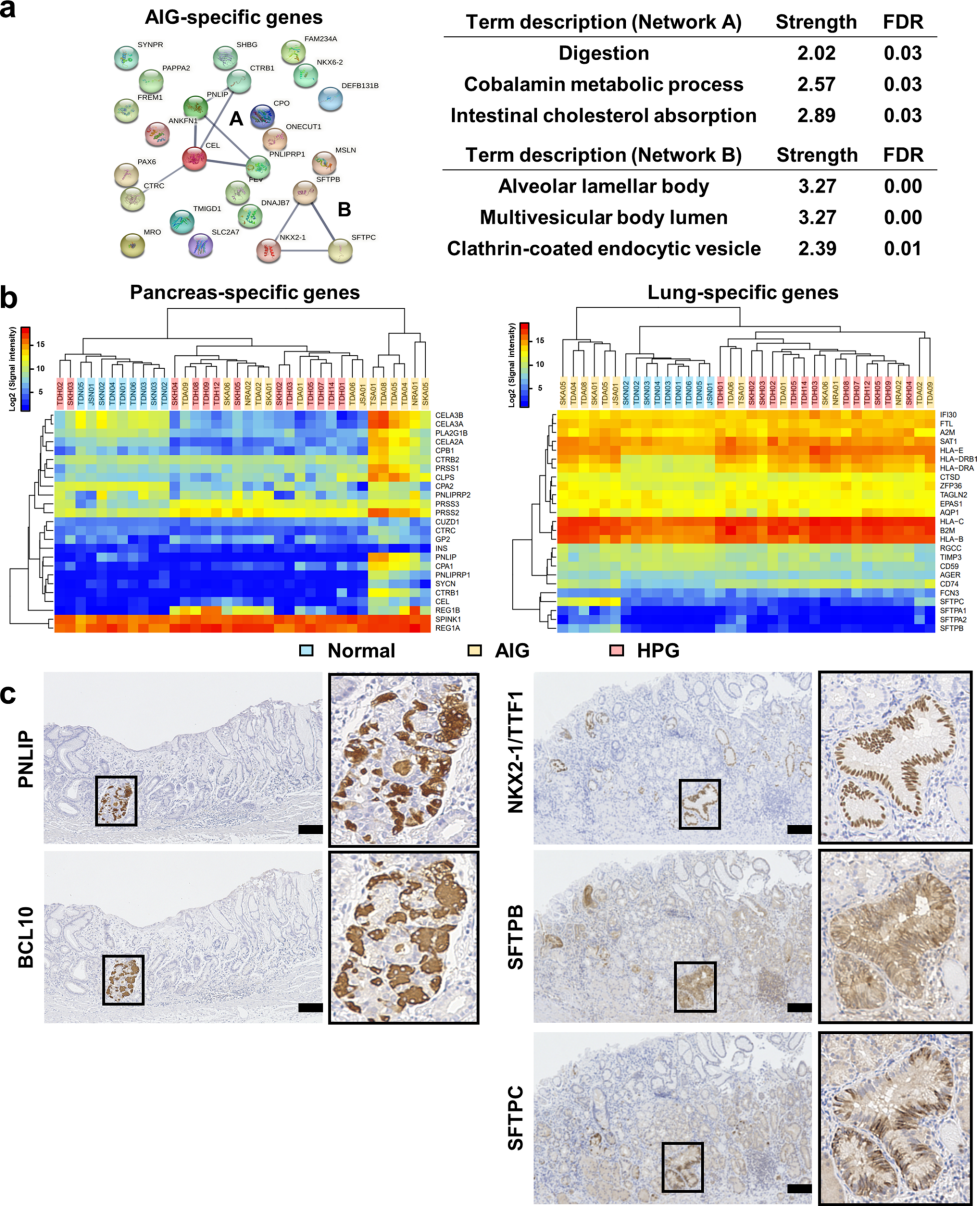
Trans-differentiation into the pancreas and lung in gastric mucosa with AIG. **a** Protein–protein interaction (PPI) network analysis using AIG-specific genes. The observed networks (Network A and Network B) showed the enrichment of gene sets related to digestion and the alveolar lamellar body. *FDR*—false discovery rate. **b** Unsupervised hierarchical cluster analysis using gene expression levels of pancreas-specific and lung-specific genes among the AIG (*n* = 14), HPG (*n* = 13), and normal samples (*n* = 9). A fraction of the AIG samples was clearly separated from the other samples. **c** Immunostaining of AIG-specific genes related to abnormal differentiation of the pancreas (PNLIP and BCL10) and lung (NKX2-1/TTF1, SFTPB, and SFTPC) using gastric mucosa with AIG. Scale bar: 100 μm

Next, we identified the top 25 upregulated genes specific to the mucosa with AIG (Fig. 4a, left), which showed low expression levels in the normal and HPG samples (average signal intensity < 25) and minimal expression changes between the normal and HPG samples (< twofold change) (Table S10). Notably, AIG-specific genes include pancreatic digestion-related genes, pancreatic triacylglycerol lipase (*PNLIP*) [21], carboxyl ester lipase (*CEL*) [22], and chymotrypsin B1/C (*CTRB1* and *CTRC*) [23]. In addition, a master regulator gene of the lung, NK2 homeobox 1/thyroid transcription factor 1 (*NKX2-1/TTF1*) [24], and alveolar fluid secretion-related genes, surfactant proteins B and C (*SFTPB* and *SFTPC*) [25], were included. Furthermore, the protein–protein interaction (PPI) network analysis using AIG-specific genes showed enrichment of gene sets related to digestion and alveolar lamellar bodies (Fig. 4a, right). A fraction of AIG samples actually expressed these genes (Figs. S3 and S4) and were clearly separated from the other samples (Fig. 4b) using the pancreas- and lung-specific genes (Tables S11 and S12). We also confirmed these ectopic gene expressions (Figs. S5 and S6) by RT-PCR on an additional sample set (Table S1).

To confirm the level and distribution of protein expression, we performed immunohistochemical analysis of the newly identified gene products in the AIG samples (Figs.4c and S7). PNLIP and BCL10, which are markers of pancreatic acinar cells [26], were co-expressed in a restricted area of the gastric mucosa with AIG, indicating that pancreatic metaplasia [27] causes ectopic pancreas-specific gene expression. In contrast, diffused expression of NKX2-1/TTF1 was observed in gastric mucosa with AIG, along with two types of surfactant proteins, SFTPB and SFTPC, suggesting that pulmonary trans-differentiation universally occurs in the stomach with AIG. Taken together, gastric mucosa with AIG undergoes more diverse trans-differentiation than that with HPG.

In addition, the upregulated genes specific to the mucosa with HPG (Table S13) included cytokines, *IL6*, *CXCL8*, and *CXCR2* and showed enrichment of multiple gene sets related to the inflammatory response (Fig. S8). *CXCL8* is mainly secreted by macrophages and induces neutrophil migration, which is in accordance with the histological findings [28] and the results of the pathway enrichment analysis described above (Fig. 2c).

### Environmental acidic condition possibly affects expression levels of intestine-related genes

To explore the impact of AIG on gene expression, we first focused on DNA methylation, as gastric mucosa with AIG showed aberrant DNA methylation at promoter CpG islands (CGI) [6]. Notably, most of the upregulated and downregulated genes had no promoter CGI (Tables S4 and S5), indicating their regulation by TATA box elements. Typically, CGI-regulated genes are crucial for fundamental cellular processes, including housekeeping and tumor suppressor genes [29–31]. In contrast, TATA-regulated genes often exhibit regulated or inducible gene expression [32], meaning their expression levels are modulated in response to specific environmental conditions and stimuli.

**Fig. 5:**
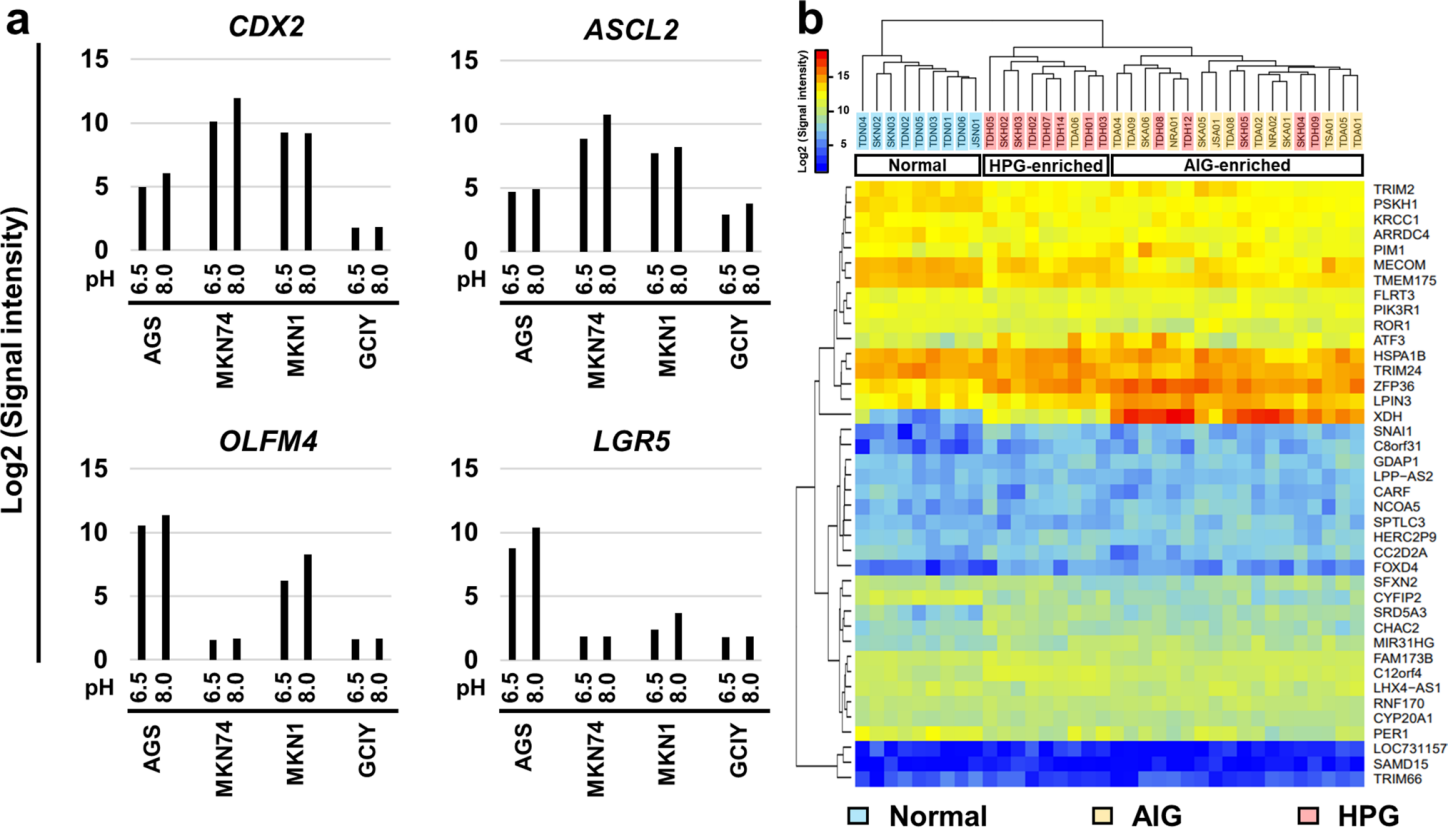
Abnormal intestinal differentiation by environmental acidic conditions. **a** Gene expression changes in gastric cancer cell lines (AGS, MKN74, MKN1, and GCIY). Acidic conditions downregulated the expression levels of a master regulator gene of the small intestine, *CDX2*, and stem marker genes, *LGR5*, *ASCL2,* and *OLFM4*. **b** Unsupervised hierarchical cluster analysis using gene expression levels of pancreas-specific and lung-specific genes among the AIG (*n* = 14), HPG (*n* = 13), and normal samples (*n* = 9). The AIG and HPG samples were clearly separated from the normal samples and were further separated into the AIG-enriched cluster and the HPG-enriched cluster

Therefore, we focused on the environmental changes predominantly observed in the gastric mucosa with AIG. Reflecting the destruction of parietal cells by AIG, the expression levels of *ATP4A* and *ATP4B* were drastically decreased in AIG samples compared with those in HPG samples (Fig. S9 and Tables S14 and S15), and a markedly high pH has been reported in the mucosa with AIG [33, 34]. Therefore, we hypothesized that an increased environmental pH might induce abnormal trans-differentiation in the gastric mucosa with AIG. To test this hypothesis, gastric cancer cells (AGS, MKN74, MKN1, and GCIY) were cultured under acidic or control conditions to analyze changes in gene expression. Remarkably, acidic conditions downregulated the expression levels of *CDX2*, a master regulator gene of the small intestine [17], and stem marker genes *LGR5* [35], *ASCL2* [36], and *OLFM4* [37] (Fig. 5a), and the average fold changes of small intestine-specific genes among the cell lines tended to be downregulated in acidic conditions (Fig. S10). In contrast, there was minimal change in the expression of pancreatic and pulmonary genes identified in the AIG.

Next, we comprehensively analyzed the genes that were upregulated and downregulated (> twofold change) under acidic conditions in each cell line and identified the commonly upregulated (*n* = 7) and downregulated genes (*n* = 33) (Fig. S11 and Table. S16). Based on these possible pH-dependent genes, the AIG and HPG samples were clearly separated from the normal samples and were further separated into the AIG-enriched cluster and the HPG-enriched cluster (Fig. 5b), suggesting that increased environmental pH may alter gene expression in the gastric mucosa, consequently resulting in abnormal differentiation in the intestine.

## Discussion

To the best of our knowledge, this is the first report of a comprehensive analysis of AIG-induced gene expression changes. Gastric mucosa with AIG shows a unique gene expression profile compared with that with HPG or non-inflammatory normal mucosa. Notably, AIG presents abnormal pancreatic and pulmonary differentiation and well-known metaplastic intestinal differentiation. Increased environmental pH owing to AIG may cause abnormal differentiation of the gastric mucosa.

Both AIG and HPG elicit ectopic intestinal differentiation as a result of long-term chronic inflammation in the stomach, and our results provide novel evidence that the gastric mucosa with AIG shows more marked expression of intestinal genes than HPG. However, HPG begins in the antrum and spreads into the gastric body, leading to intestinal metaplasia primarily in the antrum. To reduce the effect of the main “inflammation place” on gene expression differences between AIG and HPG, we aligned the mucosal condition to severe open-type atrophy between AIG and HPG cases and used the biopsy samples obtained from the middle region of the body. Consistent with the difference at the gene expression level between AIG and HPG, AIG samples exhibited a higher incidence of intestinal metaplasia histologically than the HPG samples.

Intestinal metaplasia is considered a risk factor for gastric cancer. However, this contrasts the observation that patients with AIG without *H. pylori* infection have a lower incidence of gastric cancer than those with HPG [3]. Additionally, gastric cancers developed from AIG tended to display gastric-type mucin [38]. One possibility is that the degree of inflammatory response, which induces aberrant DNA methylation [39–43] involved in carcinogenesis, is lower in the mucosa with AIG than in that with HPG. Pathway enrichment analysis using differentially expressed genes showed that the gastric mucosa with HPG was more enriched with gene sets associated with the inflammatory response of the macrophage–neutrophil axis. Gastric mucosa with HPG was reported to have more aberrant DNA methylation than that with AIG [6]. Another possibility is that *H. pylori*-associated virulent factors such as CagA play a crucial role in the development of gastric cancer [44, 45]. Alternatively, the disorder of the chromatin-remodeling factor, the SWI/SNF complex, has been implicated in gastric cancer development in patients with HPG and may appear in the gastric mucosa with HPG [46].

In contrast, AIG causes tumorigenesis of neuroendocrine tumor possibly by elevated gastrin levels due to the destruction of parietal cells. Indeed, the cases of AIG showed extremely high serum gastrin level (Tables 1 and S2), and the gastric mucosa with AIG had high expression of *GAST* and *PAPPA2* (Fig. S1), which are reported to be involved in neuroendocrine tumorigenesis [14]. Generally, *GAST* is expressed in G cells located in pyloric gland. The high expression levels in the middle region of the body might reflect the expression levels in G cells within pyloric gland metaplasia, since it has been reported that AIG induced pyloric gland metaplasia and intestinal metaplasia [2].

Some AIG samples showed ectopic expression of pancreas-related and lung-related genes, which was not observed in HPG samples. The pancreatic digestion-related genes, namely *PNLIP*, *CEL*, *CTRB1*, and *CTRC*, were upregulated, which concurs with the fact that metaplasia of pancreatic acinar cells is observed in the mucosa with end-stage AIG [2, 27, 47]. In addition, a major pancreatic transcriptional factor, *PDX1* [48], was not expressed in all the samples (signal intensity < 25), suggesting other mechanisms of ectopic expression. Pulmonary differentiation by AIG is a completely novel finding; a master regulator gene of lung, *NKX2-1/TTF1*, and alveolar fluid secretion-related genes, *SFTPB* and *SFTPC*, were also upregulated, which may cause the sticky, adherent, and dense mucus frequently observed in the gastric mucosa with AIG during endoscopic examination [49]. We have previously reported that gastric adenocarcinoma of the fundic gland type shows high expression levels of *SFTPB* and *SFTPC*, both of which are transactivated by the ectopic expression of *NKX2-1/TTF1* [50]. Ectopic expression of pancreas-related and lung-related genes was also confirmed at the protein level, revealing that gastric epithelial cells with AIG have the potential to trans-differentiate in not only the well-known intestine but also the pancreas and lung.

Regarding the mechanism of abnormal differentiation, increased environmental pH due to the destruction of parietal cells could affect gene expression in the gastric mucosa with AIG [33, 34]. Both AIG and HPG showed the downregulation of *ATP4A* and an increase in environmental pH, but the alleviation in acidic conditions was much more prominent in the gastric mucosa with AIG. Notably, gastric cancer cell lines, which often express *CDX2* and other intestine-related genes, were downregulated under low pH conditions. Therefore, environmental changes in pH may be a potential mechanism for the abnormal differentiation of gastric mucosa with AIG. This hypothesis is intriguing, as the environmental pH in the gastric mucosa with HPG is also elevated owing to *H. pylori*-induced chronic inflammation of the stomach [51]. A significantly elevated environmental pH may be the initial step in the unstable differentiation of the gastric epithelium.

There are certain limitations and a need for the future trial concerning our study. First, only a limited number of samples were analyzed by immunohistochemistry for AIG-specific genes. To evaluate their potential as valuable auxiliary diagnostic markers to identify AIG patients or as risk markers for tumorigenesis, a large cohort or prospective study is required. Second, many types of gastric and inflammatory cells in biopsy samples hinder the assessment of the expression profiles and signaling pathways. Single-cell analysis is required to more accurately analyze the impact of AIG on gene regulation according to cell types and to understand the mechanisms underlying abnormal differentiation and tumorigenesis, including that of NET. Third, even if AIG and normal cases are* H. pylori*-negative with no eradication history, it is difficult to strictly exclude the possibility of past *H. pylori* infection. A novel diagnostic modality is required. Finally, the effects of the environmental pH on gene expression were assessed in gastric cancer cell lines. To further enhance our analysis, normal gastric epithelial cells or organoids should be considered.

In conclusion, AIG induced diverse trans-differentiation characterized by the transactivation of genes specific to the small intestine, pancreas, and lung. Increased environmental pH caused by AIG may lead to abnormal differentiation of the gastric mucosa.

### Supplementary Information

Below is the link to the electronic supplementary material.

Supplementary file1 (DOCX 2162 KB)

Supplementary file2 (XLSX 69 KB)
