# Supplementary material for: Marked intestinal trans-differentiation by autoimmune gastritis along with ectopic pancreatic and pulmonary trans-differentiation
Source: J Gastroenterol. 2023 Nov 14;59(2):95–108. doi: 10.1007/s00535-023-02055-x (PMC10810929; doi:10.1007/s00535-023-02055-x)
Supplement: Supplementary file 1 — Supplementary file1 (DOCX 2162 KB) [file 535_2023_2055_MOESM1_ESM.docx]

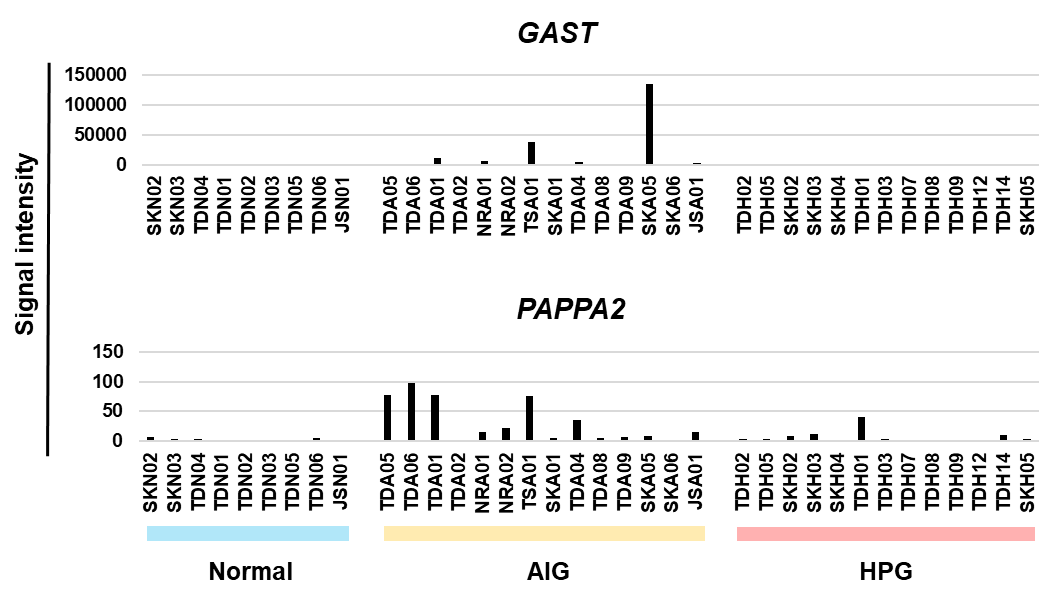


## Fig. S1

Gene expression levels of *GAST* and *PAPPA2* among the AIG (n = 14), HPG (n = 13), and normal samples (n = 9). *GAST* and *PAPPA2* were highly expressed in the AIG samples.


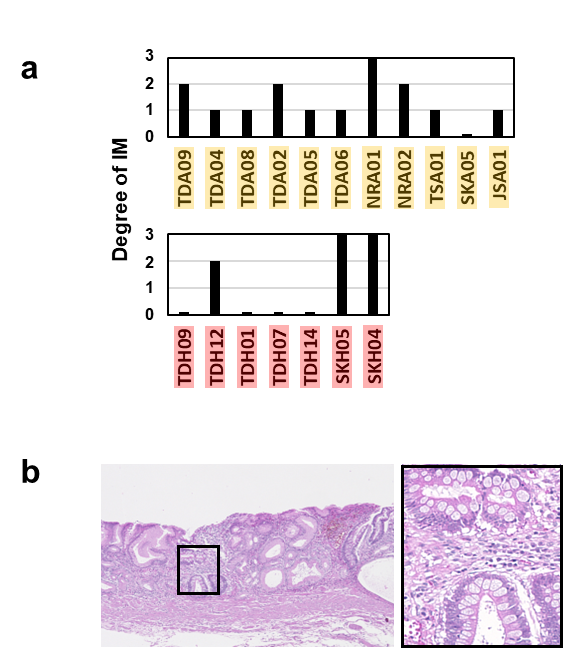


## Fig. S2

Marked intestinal differentiation in gastric mucosa with AIG. (a) Histological analysis of the AIG (n = 11) and HPG samples (n = 7) based on the Updated Sydney system (USS). Gastric mucosa with AIG showed a higher incidence of intestinal metaplasia than that with HPG. The USS scores were graded on a scale of 0–3 (none, 0; mild, 1; moderate, 2; severe, 3). (b) H&E staining using gastric mucosa with AIG. Scale bar: 100 μm.


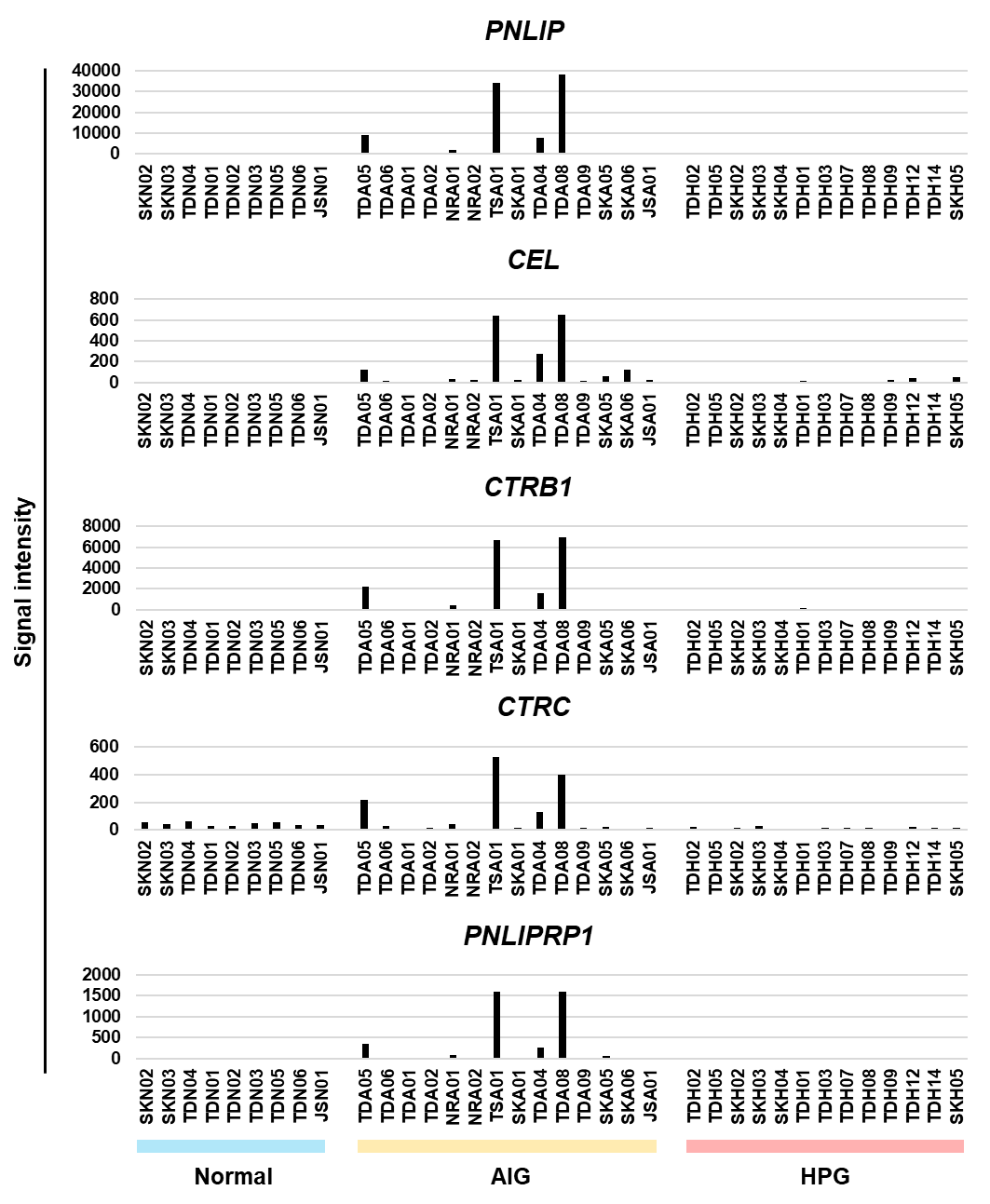


## Fig. S3

Gene expression levels of AIG-specific genes among the AIG (n = 14), HPG (n = 13), and normal samples (n = 9). A fraction of the AIG samples showed ectopic expression of pancreatic digestion-related genes, *PNLIP, CEL, CTRB1*, *CTRC*, and *PNLIPRP*.


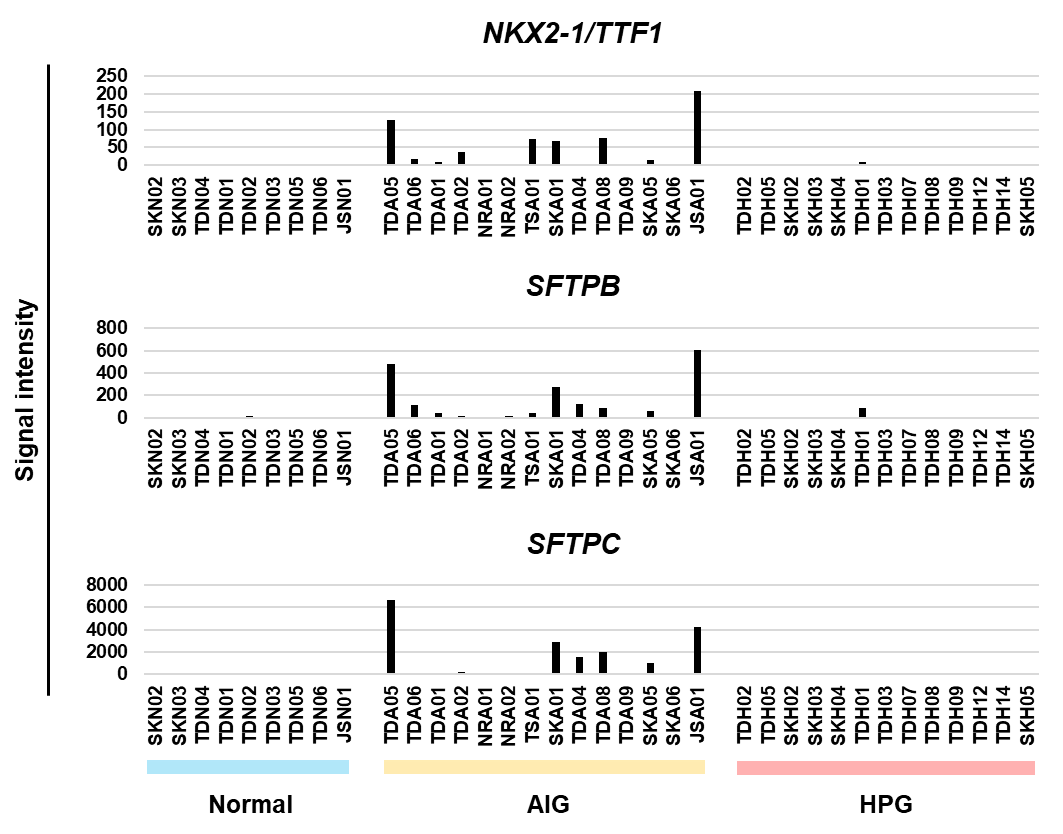


## Fig. S4

Gene expression levels of AIG-specific genes among the AIG (n = 14), HPG (n = 13), and normal samples (n = 9). A fraction of the AIG samples showed ectopic expression of a master regulator gene of lung, *NKX2-1/TTF1*, and alveolar fluid secretion-related genes, *SFTPB* and *SFTPC*.


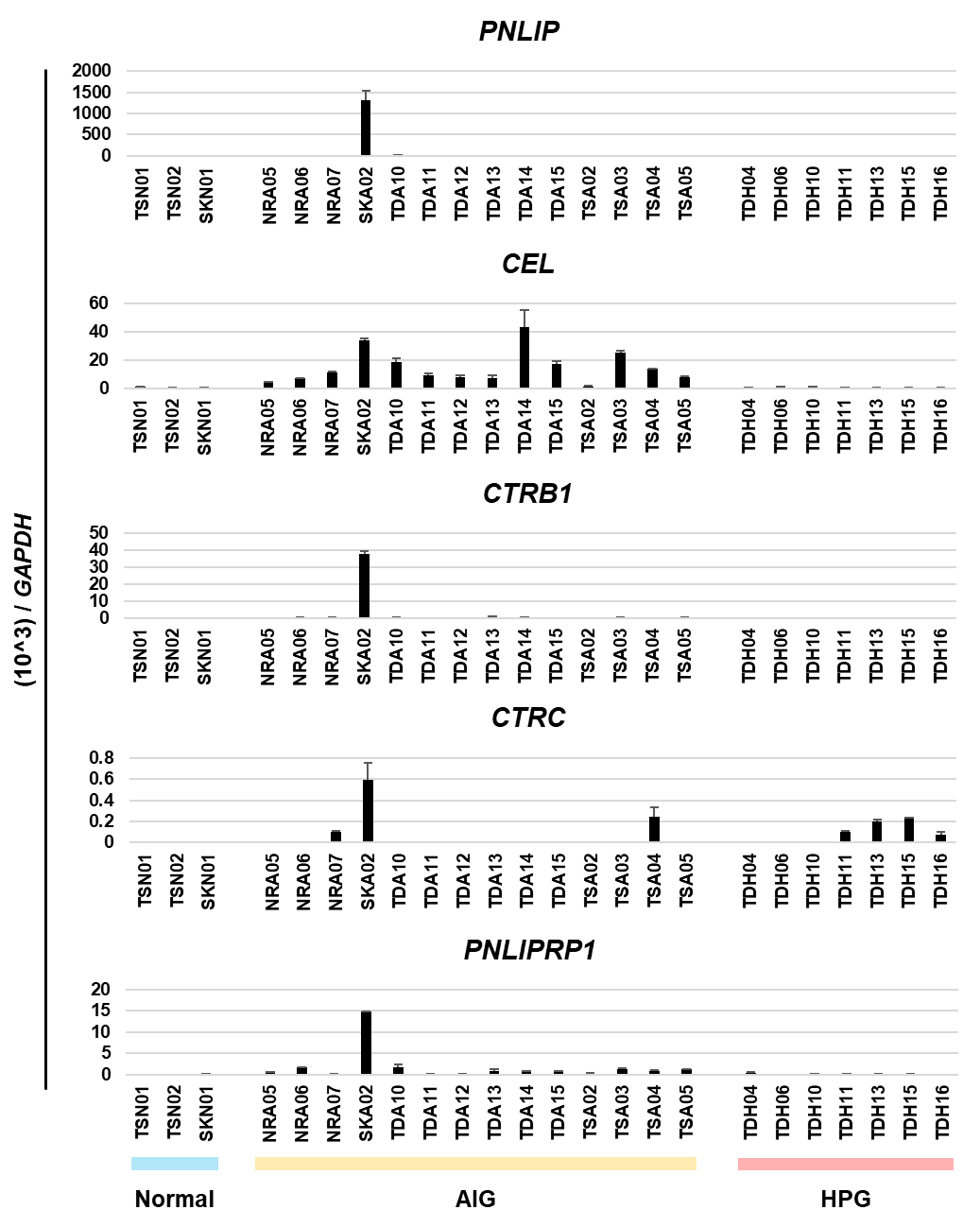


## Fig. S5

Gene expression levels of AIG-specific genes in an additional sample set, including AIG (n = 14), HPG (n = 7), and normal samples (n = 3). A fraction of the AIG samples showed ectopic expression of pancreatic digestion-related genes, *PNLIP, CEL, CTRB1*, *CTRC*, and *PNLIPRP*.


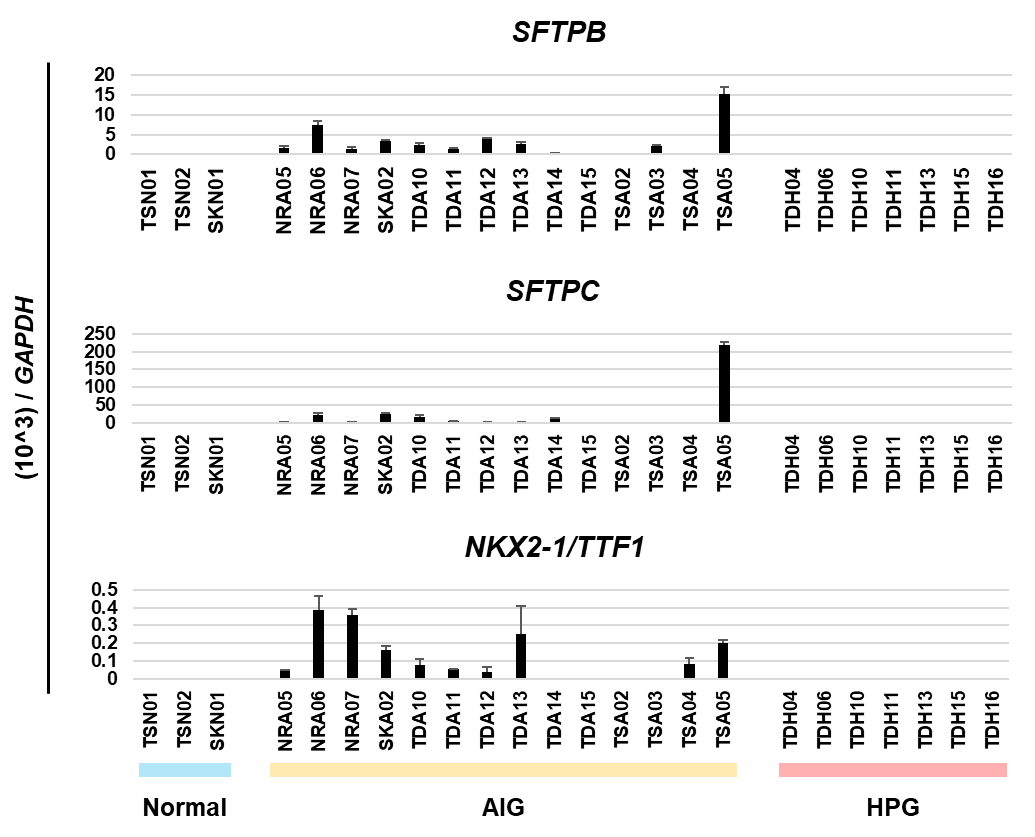


## Fig. S6

Gene expression levels of AIG-specific genes in an additional sample set, including AIG (n = 14), HPG (n = 7), and normal samples (n = 3). A fraction of the AIG samples showed ectopic expression of a master regulator gene of the lung, *NKX2-1/TTF1*, and alveolar fluid secretion-related genes, *SFTPB* and *SFTPC*.


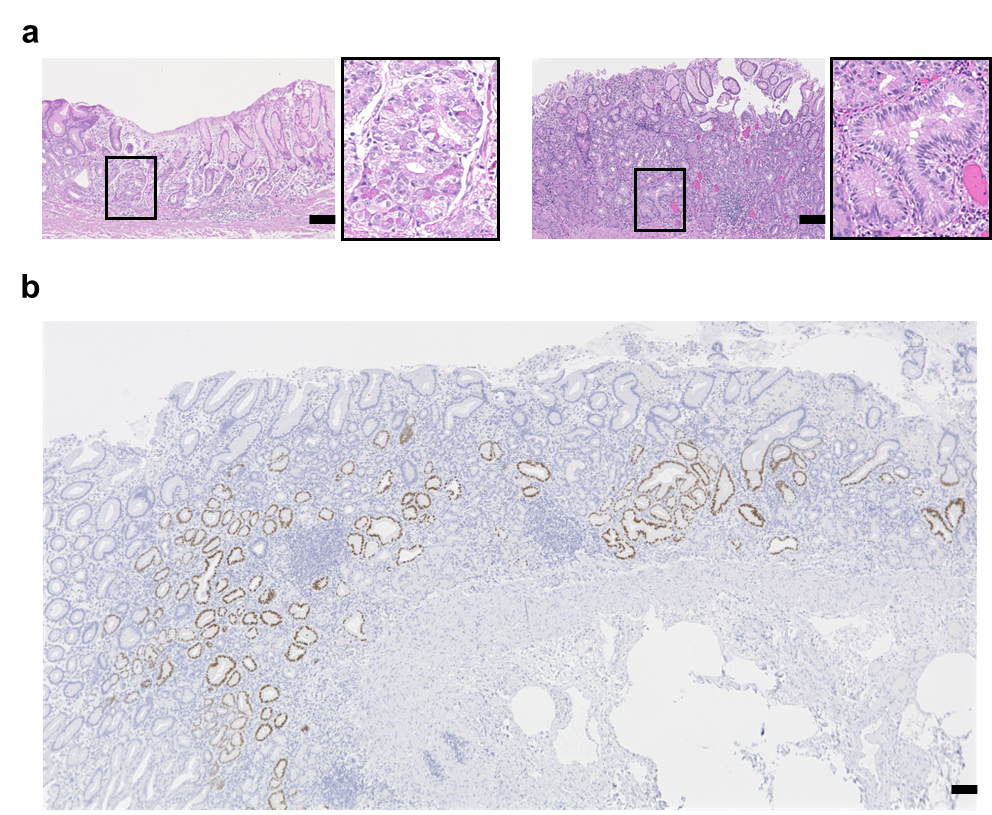


## Fig. S7

Trans-differentiation into the pancreas and lung in gastric mucosa with AIG. (a) The images of H&E staining corresponding to the Fig. 4c. (b) Diffuse staining of NKX2-1/TTF1 in gastric mucosa with AIG. Scale bar: 100 μm.


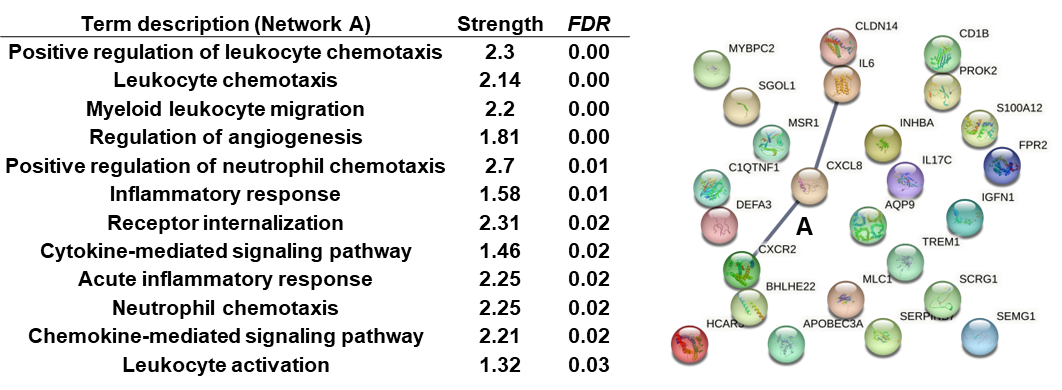


## Fig. S8

Protein–protein interaction (PPI) network analysis using HPG-specific genes. The upregulated genes specific to the mucosa with HPG included cytokines, *IL6*, *CXCL8,* and *CXCR2*, and showed enrichment of a large number of gene sets related to inflammatory response. FDR = false discovery rate


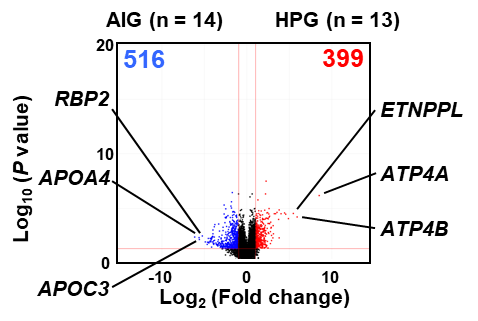


## Fig. S9

Volcano plot analysis using the fold changes of gene expression levels between the AIG (n = 14) and HPG (n = 13) samples. The expression levels of *ATP4A* and *ATP4B* were drastically decreased in the AIG samples compared with those in the HPG samples.


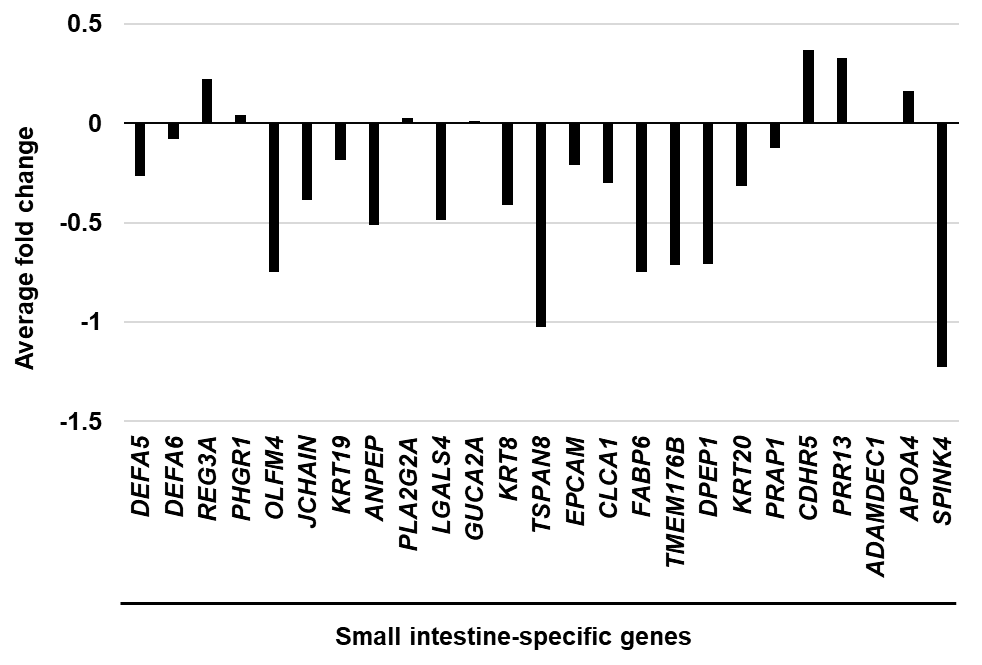


## Fig. S10

Gene expression changes of small intestine-specific genes in acidic conditions. The average fold changes among the cell lines tended to be downregulated in acidic conditions.


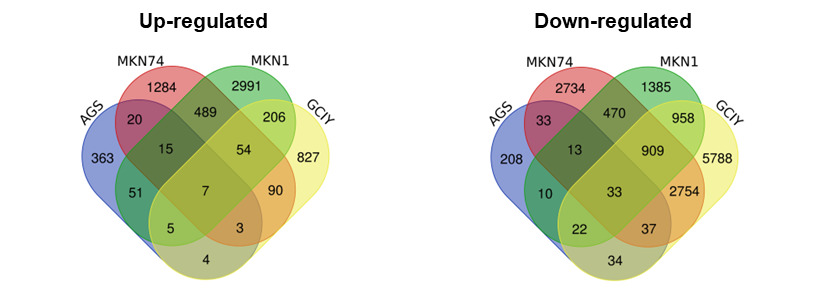


## Fig. S11

Abnormal intestinal differentiation by environmental acidic condition. The common upregulated and downregulated genes among gastric cancer cell lines (AGS, MKN74, MKN1 and GCIY).
